# Tribute to Professor McDowell

**Published:** 1835

**Authors:** 


### Art. III.—Tribute of respect to Professor McDowell.

The dissecting class of the Cincinnati College, in the month of February, presented to Dr. McDowell, a beautiful silver vase, with a suitable inscription. We did not witness the little ceremony, which took place in the anatomical theatre, but have understood, that Mr. Johns, of Tennessee, pronounced on the occasion, in behalf of his fellow students, a chaste and appropriate address, which was answered by the young professor with his characteristic fervency.

